# The influence of subcolony-scale nesting habitat on the reproductive success of Adélie penguins

**DOI:** 10.1038/s41598-021-94861-7

**Published:** 2021-07-28

**Authors:** Annie E. Schmidt, Grant Ballard, Amélie Lescroël, Katie M. Dugger, Dennis Jongsomjit, Megan L. Elrod, David G. Ainley

**Affiliations:** 1 Point Blue Conservation Science, Petaluma, CA 94954 USA; 2 U.S. Geological Survey, Oregon Cooperative Fish and Wildlife Research Unit, Department of Fisheries and Wildlife, Oregon State University, Corvallis, OR 97331 USA; 3 H.T. Harvey & Associates Ecological Consultants, Los Gatos, CA 95032 USA

**Keywords:** Biogeography, Climate-change ecology, Conservation biology

## Abstract

Group-size variation is common in colonially breeding species, including seabirds, whose breeding colonies can vary in size by several orders of magnitude. Seabirds are some of the most threatened marine taxa and understanding the drivers of colony size variation is more important than ever. Reproductive success is an important demographic parameter that can impact colony size, and it varies in association with a number of factors, including nesting habitat quality. Within colonies, seabirds often aggregate into distinct groups or subcolonies that may vary in quality. We used data from two colonies of Adélie penguins 73 km apart on Ross Island, Antarctica, one large and one small to investigate (1) How subcolony habitat characteristics influence reproductive success and (2) How these relationships differ at a small (Cape Royds) and large (Cape Crozier) colony with different terrain characteristics. Subcolonies were characterized using terrain attributes (elevation, slope aspect, slope steepness, wind shelter, flow accumulation), as well group characteristics (area/size, perimeter-to-area ratio, and proximity to nest predators). Reproductive success was higher and less variable at the larger colony while subcolony characteristics explained more of the variance in reproductive success at the small colony. The most important variable influencing subcolony quality at both colonies was perimeter-to-area ratio, likely reflecting the importance of nest predation by south polar skuas along subcolony edges. The small colony contained a higher proportion of edge nests thus higher potential impact from skua nest predation. Stochastic environmental events may facilitate smaller colonies becoming “trapped” by nest predation: a rapid decline in the number of breeding individuals may increase the proportion of edge nests, leading to higher relative nest predation and hindering population recovery. Several terrain covariates were retained in the final models but which variables, the shapes of the relationships, and importance varied between colonies.

## Introduction

For colonially breeding birds, variation in the size of breeding aggregations is pervasive^1–3^. Many seabird species form colonies that range in size by several orders of magnitude^4^. Despite decades of investigation^e.g.^^1,5–7^, understanding the causes and consequences of this variability remains an important topic of research. Some colonies grow while others do not^8^ and colonies of different sizes may face unique challenges (e.g., increased competition and/or predation). Seabirds are one of the most threatened marine taxa and the most threatened group of birds^9^ with monitored species declining nearly 70% between 1950 and 2010^10^. In an era of rapid climate and ecosystem change, understanding the conditions under which seabird colonies grow and/or recover from environmental disturbance is more important than ever.

Reproductive success is an important demographic parameter influencing population dynamics that has been well studied in seabirds. Numerous factors can influence the reproductive output of a colony^11^, including prey availability^12–14^, density dependent competition^5^, and nest predation^6^. Because seabirds are typically on the slow end of the life-history continuum (i.e., long-lived with lower reproductive rates), population growth is often more sensitive to changes in adult and subadult survival than reproductive success^15^. Nonetheless, lower reproductive success can depress population growth and sustained low reproductive success can lead to a higher risk of population decline or extinction^16,17^.

Variability in nesting habitat quality, both within and between colonies, can be an important driver of seabird reproductive success and may contribute to differences in colony growth and ultimately size. For example, Common murre (*Uria aalge*) individuals are more successful when nesting on shallow slopes protected by walls^18^, Yellow-legged gulls (*Larus michahellis*) have higher success in vegetated versus bare habitat^19^, similar to Magellanic penguins (*Spheniscus magellanicus*) who showed higher reproductive success in nest sites with higher vegetation cover^20^. For European shags (*Phalacrocorax aristotelis*), reproductive success is related to overhead cover, drainage, and visibility^21^. In several of these examples, the inferred cause of nest failure was often predation, with variability in nesting habitat quality ultimately determined by how visible or accessible an area was to nest predators.

Defense against predation is considered to be an advantage of colonial breeding, with greater predator defense or predator swamping occurring in larger aggregations^22^. Colonies are frequently composed of smaller groups of contiguously nesting birds of various size and shapes (subcolonies), and nest predation is typically higher at the edges of subcolonies^23–28^. The higher risk of predation along the edges of animal aggregations is a characteristic of the “selfish herd” concept in which the tendency to aggregate is selected for as individuals attempt to minimize individual risk by closely associating with conspecifics^29^. However, direct evidence linking nest predation pressure and colony size is somewhat equivocal, with predation both increasing^25,30^ and decreasing^7^ with colony size.

The Adélie penguin (*Pygoscelis adeliae*) is one of two penguin species that breeds only in Antarctica and the global distribution of their colony sizes is well described^4,31,32^. A majority of Adélie colonies are relatively small, with most containing < 3000 pairs each and fewer than 10 colonies containing > 100,000 pairs^31,33^. Colonies tend to occur in clusters, with small colonies clustered around a large one^4^. Adélie penguins are dependent on sea ice, a habitat that has been rapidly changing in some regions of Antarctica and expected to continue to change in the future^34–36^. Many colonies on the Antarctic Peninsula, in the northern part of the Adélie range, are already decreasing with declines projected to continue under future climate scenarios^36,37^. Studies from the Peninsular region indicate that variability in nesting habitat quality may be contributing to population declines at some colonies through its influence on nesting success^38–41^. For example, the population trends at many colonies are related to the amount of “suboptimal” habitat, characterized as having a southwest aspect where snow is more likely to accumulate^38,40^. Colonies that are more prone to early season snow accumulation also exhibit later breeding phenology and lower reproductive success^39,41^. The vulnerability to snow accumulation at some colonies is particularly relevant as snowfall is predicted to increase as climate warms^36^.

On Ross Island, located in the Ross Sea and at the southern edge of Adélie penguin breeding range, a metapopulation consisting of four colonies (including one on Beaufort Island) of varying sizes has been intensively studied for several decades^8,32,42^. The smallest colony in the cluster, Cape Royds, has varied between 2000 and 4000 breeding pairs^8,27,43^, comparable with the majority of Adélie colonies. On the other hand, the largest colony in the cluster, Cape Crozier, is one of the largest colonies of the species^31,33^. Over the last two decades, the number of breeding pairs in the Cape Crozier colony has grown steadily, despite showing evidence of higher competition and negative density dependence (e.g. longer foraging trips and smaller chicks^44–47^). While to some degree the differences in population trends may be the result of external forcing, such as unreliable access to sufficient open water at Cape Royds^43^, or variability in prey availability and ocean conditions^48^, they suggest a need to better understand the various factors contributing to differential population growth, including the role of nesting habitat quality.

Warming is expected to increase the amount of ice-free habitat available continent wide^49^, which may increase the amount of available nesting habitat, but whether the newly exposed habitat is colonized may depend on the relative quality of the newly exposed areas. Although glacial retreat has been associated with colony expansion on Beaufort Island^50^, the Ross Island Adélie colonies are the farthest south for the species and likely represent an important climate refugia, buffered from the worst impacts of warming (e.g. sea ice retreat) but also experiencing less “benefit” from new ice-free habitat^36,49^.

Here we use data on the annual reproductive success of penguins nesting in subcolonies with different habitat characteristics over 4 years at two Ross Island colonies separated by 73 km to investigate the following: (1) How do subcolony habitat characteristics influence reproductive success? and (2) How do these relationships differ at a small (Cape Royds) and large (Cape Crozier) colony with different terrain characteristics? The Ross Island metapopulation contains ~ 10% of the global Adélie penguin population^31^, improving our understanding of the factors contributing to differential colony growth will help us anticipate impacts to this important region in the future.

## Methods

### Study sites

**Figure 1:**
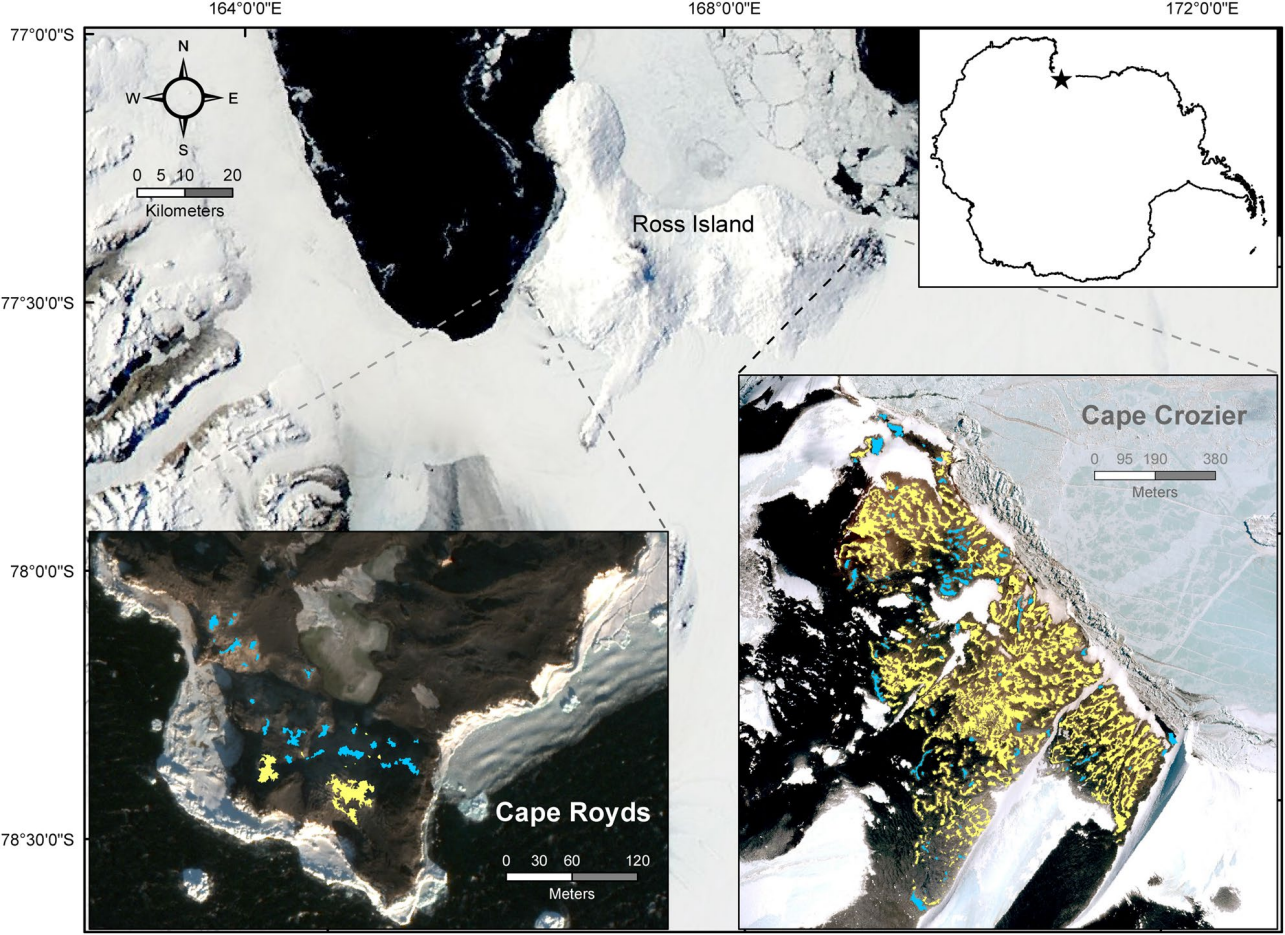
Study areas on Ross Island, Antarctica. The star on the inset map of Antarctica indicates the area of detail. Yellow polygons show all subcolonies at Cape Crozier and Cape Royds, blue polygons indicate the subcolonies used in this study to model reproductive success. Satellite image of Cape Crozier from WorldView-3, November 20, 2014 (copyright 2014 DigitalGlobe, NextView License) and of Cape Royds from WorldView-2 November 27, 2015 (copyright 2015 DigitalGlobe, NextView License). Base satellite image of Ross Island vicinity acquired by the Moderate Resolution Imaging Spectroradiometer (MODIS) on board the Aqua satellite on November 29, 2011 (courtesy of Rapid Response Imagery from the Land, Atmosphere Near real-time Capability for Earth Observing System operated by the NASA/GSFC/Earth Science Data and Information System with funding provided by NASA/HQ).

The study was conducted on two colonies on Ross Island, Cape Crozier (77°27′S, 169°14′E) and Cape Royds (77°33′S, 166°10′, Fig. 1). Breeders arrive at Ross Island in late October/early November, build a nest out of pebbles, and lay a maximum of two eggs by mid-November^32,42^. Nests are distributed in distinct subcolonies, often on mounds and ridges created or enhanced by years of nest stone and guano deposition^51,52^. Individual territories average approximately 0.75 m^2^^53,54^. Chicks hatch about 35 days after egg laying and are fed by both parents from mid-December to early February before fledging^32^. Chicks form groups (créches) after ~ 14 days if both parents are away foraging^32^. We investigated the effect of subcolony habitat characteristics on annual reproductive success over 4 breeding seasons, spanning the austral summers from 2014–2015 through 2017–2018. Hereafter, we refer to austral summers as seasons, using their initial year (e.g., 2014 refers to the 2014–2015 breeding season).

### Annual reproductive success by subcolony: mean chicks per pair

Ground counts of nests and chicks have been conducted annually at Cape Crozier and Cape Royds since 1997^47^. A consistent subset of 27 subcolonies (out of > 700 total) within a core study area were counted every year at Cape Crozier beginning in 2002 while a majority (21–22 of ~ 30) of the subcolonies have been counted annually at Cape Royds. To generate a more representative sample of the whole Cape Crozier, we counted an additional 29–56 subcolonies distributed throughout the colony per season for the purposes of this study (2014–2017, Table 1). These additional subcolonies were selected using a stratified random sample based on hypothesized key attributes (see next section). Counting from the ground can only be done for relatively small subcolonies so a number of larger subcolonies (> 1000 active territories) that could be counted via photographs were added to the study at Cape Crozier in 2016. At both colonies, the number of active territories (nests with eggs) in each study subcolony was counted in early December, coinciding with peak incubation and a period when there were few non-breeders present. Subsequently, each study subcolony was revisited in mid-January approximately 1 week after the median créche date (the date when half of the chicks had entered the créche), to count the total number of chicks present (guarded or crèched). The annual reproductive success of each subcolony was estimated by dividing the total number of chicks counted by the initial number of active territories to derive the mean number of chicks produced per breeding pair. Mean reproductive success over the 4-year study period was compared to the whole time series (beginning in 2002) and between colonies using a generalized least squares model accounting for autocorrelation and repeated measures.

**Table 1:** The number of subcolonies included in the subcolony quality model each year at Cape Crozier and Cape Royds, Ross Island, Antarctica.

Season	*C. Crozier*	*C. Royds*
2014	59	22
2015	63	22
2016	87	21
2017	83	21
Number of subcolonies counted in all 4 years	38	20

### Subcolony characteristics

We chose eight spatial attributes of subcolonies that we hypothesized to be important measures of nesting habitat quality. These included five terrain attributes, elevation, slope aspect, slope steepness, snow drift risk, and flow accumulation, as well as three other characteristics of nesting aggregations, area of subcolony, perimeter-to-area ratio, and proximity to nesting skuas. Elevation and slope aspect were included as factors that may influence exposure to stressors like heat, wind, and snowfall. Slope steepness (degrees) was also included for its potential influence on the probability of nest failure by affecting nest size, flow accumulation, and/or the probability of eggs rolling from the nest^55^. Excessive snow drift can cause nest failures by burying nesting penguins and melting snow can chill or drown eggs and chicks^38,41^. We estimated exposure and snow drift risk by calculating a wind shelter index^56,57^ and a flood risk index from surface flow accumulation for each subcolony (see below for description of methods for these variables). The area covered by the subcolony and the number of penguins nesting in the subcolony are highly correlated (r = 0.98, p << 0.001 for subcolonies in this study) so we included just the area (m^2^) of each subcolony among the factors influencing reproductive success and included the perimeter-to-area ratio (m/m^2^) to index the relative number of edge nests versus interior nests. Finally, south polar skuas (*Stercorarius maccormicki*), which nest in and around Adélie colonies, defend foraging territories, including penguin subcolonies, which are anchored by their nest site, but which vary in size and shape^58,59^. Penguins that nest within a foraging territory of a skua, may experience different nest predation pressure^59^ than other nesting penguins. We did not have data on the size of specific foraging territories so we included whether or not a subcolony had at least one skua pair nesting nearby (within 50 m of the nearest edge) as a binary factor in the model.

Spatial attributes of subcolonies were calculated using ArcGIS (ESRI ArcGIS Desktop v10.5.1 and 10.7.1) and program R (v.3.6.1, R Core Development Team, 2019). Subcolony boundaries were established by digitizing the outlines of nesting groups from aerial images taken from a helicopter ~ 700 m above ground level in 2014 (images collected and provided by Antarctica New Zealand). Images were first aligned using Photoscan Pro (Agisoft Photoscan Pro v1.6.2) to generate a point cloud, from which an orthorectified mosaicked image was created. The image mosaics were then georeferenced in ArcGIS using a satellite image (for Cape Crozier, WorldView-3 image date November 20, 2014, for Cape Royds WorldView-2 image date November 27, 2015; Copyright DigitalGlobe, NextView License) and several ground control points collected at landmarks for spatial reference. In a few cases at Cape Crozier, an image could not be placed in the mosaic via the automated process and was subsequently georeferenced manually to obvious landmarks in the mosaic (14 out of 104 images placed manually). Images from 2009 (the last time complete photographic coverage of Cape Crozier was achieved) were used to fill in any remaining holes (< 3% of the total colony area). Subcolony boundaries were traced from the complete mosaic by placing a polygon vertex on each perimeter nest visible in the aerial photos with typically no more than 1.5 m between each vertex. Subcolonies were defined as discrete patches of at least 3 pairs of contiguously nesting penguins, with at least 1.5 m separating any adjacent subcolonies. The smallest subcolony included in the analysis was 5 pairs at Cape Crozier and 3 pairs at Cape Royds. Subcolony area and perimeter-to-area ratio were calculated for each subcolony polygon.

The remaining terrain attributes (elevation, slope, slope aspect, wind shelter, and flow accumulation) were calculated using a digital elevation model (DEM) with 2 × 2 m grid cells and ~ 1 m vertical accuracy derived from Digital Globe Inc. imagery and created by the Polar Geospatial Center. We generated a raster layer with 2 × 2 m grid cells for each of these terrain covariates for each colony. Grid cells contained within the boundaries of each subcolony were averaged to get the mean value per subcolony of each covariate (i.e., each subcolony was assigned the average slope/aspect/elevation etc. of all the 2 m pixels contained in that subcolony).

Slope aspect calculated using GIS methods can be inaccurate near the poles where grid north and geographic north can be very different^44^. To obtain a corrected aspect map, we subtracted the longitude of each grid cell from the GIS-calculated aspect and used the equation: $longitude = arctan (x/y)$ to convert ESRI-determined aspect to values referenced to geographic north. In this equation, x and y are the longitude and latitude (respectively) of the data point after the removal of false easting and false northing^60^. Calculating the arithmetic mean aspect of subcolonies that span North (i.e., subcolonies that contain cells close to 0 degrees and cells close to 360) is inaccurate so we first calculated the cosine and sine of the aspect raster (after converting to radians), then calculated the arithmetic mean sine and cosine for every subcolony and used the two-argument arctangent function (ArcGIS atan2) to calculate the mean aspect.

An index of flood risk was generated by using the flow accumulation tool in ArcGIS. The index was calculated by first using the tool to create a raster surface of flow direction from each cell to its steepest downslope neighbor from the DEM and then accumulating the weight for all cells that flow into each downslope cell. Large snow fields (locations of which are persistent across years) were digitized from the 2014 aerial image mosaic. Upslope cells in the watershed that were covered by persistent snow were assumed to contribute more to flood risk so received double weight in the flow accumulation calculation. The raw values were log-transformed prior to analysis.

We calculated a wind shelter index to represent snow drift risk, using an algorithm implemented in program R (package RSAGA v. 1.2.0). The wind shelter calculation required the DEM and a user-supplied wind angle, tolerance, and radius. The dominant wind direction for storms at Ross Island penguin colonies is from the south with some variance between southeast^27^ and southwest^61^. We chose a wind angle of 180° (S) and a tolerance of 22.5° so that wind direction ranged from South-southeast (157.5°) to South-southwest (202.5°). A maximum search radius of 300 m defined the upwind window for each cell. Using the DEM to characterize the landscape features within the search radius, negative values of the resulting index represent low wind shelter/higher wind speeds for a given cell, while positive values represent higher shelter/lower wind speed for a cell and correspond to increased snow drift risk^56,57^.

To account for the influence of skua nest proximity on subcolony reproductive success, we mapped all skua nests within and adjacent to the Adélie colonies and along the boundary of the colony in the 2016–2017 breeding season. Skua nest locations were consistent at both colonies over the study period (visual observation), so the same nest map was used for all four breeding seasons. Distributions of subcolony characteristics at Cape Crozier and Cape Royds were compared using a Mann Whitney-U tests for non-parametric data or Chi-squared test (for proportional data). Distributions of slope aspect were compared using circular means (R package “circular”) and Watson’s two-sample test of homogeneity^62,63^.

### Modeling subcolony quality

Average reproductive success varies by colony and year^47^. To focus on spatial variability in reproductive success between subcolonies within Cape Crozier and Cape Royds, we first subtracted the annual mean number of chicks crèched per pair for the entire colony (mean from all counted subcolonies) from the mean chicks per pair produced by each subcolony. The resultant residual mean annual reproductive success, or the performance of each subcolony relative to the annual mean for the colony, was used as the dependent variable in all models. We used generalized additive models^64^ with thin-plate regression splines and included smooths for area, perimeter-to-area ratio, slope, elevation, slope aspect, wind shelter, and flow accumulation, and a binomial factor for ≥ 1 skua nest within 50 m. Variable selection was carried out automatically using select = TRUE option within the gam function which allowed variables to be dropped automatically when the smoothing parameters tended towards infinity^65^. We modeled subcolony reproductive success from Cape Crozier and Cape Royds separately and limited the basis dimension for Cape Crozier to k = 6 and for Cape Royds to k = 4 to avoid overfitting with the relatively small sample sizes, particularly at Cape Royds. Models were fit using maximum likelihood and all covariates were standardized so that the mean = 0 and SD = 1 for each colony prior to model fitting. All variables were checked for collinearity and pairwise concurvity (R package mgcv) and included in the model if both were < 0.6. At Cape Royds, slope aspect and the wind shelter index were highly correlated (r = − 0.74) so we fit models with each variable separately. Aspect is a circular variable (− 180 = 180) so the fitted smooth was constrained to meet at the ends so that the highest and lowest values of the estimated smooth were equal. We compared models using the Akaike Information Criterion corrected for small sample sizes (AICc, number of observations/number of parameters < 40 for all models^66^).

To examine whether the influence of nesting habitat covariates on reproductive success varied by year, we fit a model with a factor smooth interaction between the terrain covariates (aspect, slope, flow accumulation, wind shelter, elevation) and year so that a different smooth was generated for each year. At Cape Royds, the year interaction was added to the highest ranked model from the first step (the model including either aspect or wind shelter). Automatic variable selection was also applied to this model so that unimportant smooths could be penalized to zero. For variables retained in the top model, we estimated variable importance by removing one variable at a time, recalculating the adjusted R^2^ for the reduced model and calculating the percent difference between the top model and the reduced model. Smooths in the reduced model were constrained by passing smoothing parameters estimated in the full model to the reduced model for the remaining variables^64^.

To map predicted subcolony “quality” for all subcolonies, we used the top model (the one with no year interactions) to predict the residual mean reproductive success (subcolony quality) of all subcolonies at both Cape Crozier and Cape Royds.

## Results

### Annual reproductive success—chicks per pair

**Figure 2:**
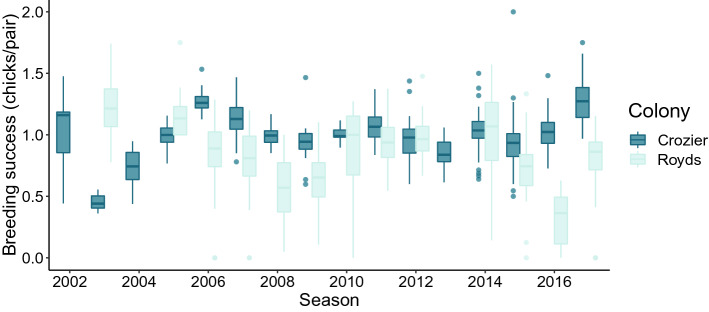
Boxplots showing annual reproductive success at Cape Crozier and Cape Royds, Ross Island, Antarctica, 2002–2017. Estimates are based on subcolony counts, the midline of the box is the median subcolony estimate with upper and lower hinges of the box representing the 25th and 75th percentiles and the whiskers extend from the hinges to the largest value no more than 1.5 times the distance between the hinges. Data beyond the ends of the whiskers are outlying points and plotted individually.

The mean number of chicks produced per pair per subcolony was higher at Cape Crozier than at Cape Royds for both the long-term time series (2002–2017) and the 4 years of this study (2014–2017; Fig. 2, Table 2). A generalized least squares model indicated that differences in mean chicks per pair between colonies over the whole time series were both significant (Table 2). At Cape Crozier, the last 4 years of the study had significantly higher reproductive success than the mean prior to the start of the study, while at Cape Royds, the 4 years of the study were significantly lower than the prior mean (Table 2). Interannual variability in reproductive success was lower at Cape Crozier (coefficient of variation CV_an_ = standard deviation (sd) of annual mean reproductive success divided by mean reproductive success of all subcolonies in all years, 2002–2017, CV_an_ = 0.20) compared to Cape Royds (CV_an_ = 0.30). Within-year spatial variability was also lower at Cape Crozier (mean CV_sp_ = sd of estimated reproductive success of subcolonies within a year divided by the mean for that year, CV_sp_ = 0.15 for 2002–2017) compared to Cape Royds (mean CV_sp_ = 0.38 for 2002–2017).

**Table 2:** Differences in reproductive success between colonies, Cape Crozier and Cape Royds, Ross Island, Antarctica, and time periods estimated with a generalized least squares model accounting for autocorrelation and repeated measures at subcolonies.

Model	Colony	Period	Estimated mean	t	p
1	C. Crozier	2002–2017	1.04 ± 0.02	67.54	< 0.001
	C. Royds	2002–2017	0.83 ± 0.03	− 7.66	< 0.001
2	C. Crozier	2014–2017	1.08 ± 0.02	64.28	< 0.001
	C. Royds	2014–2017	0.72 ± 0.04	− 10.19	< 0.001
3	C. Crozier	2002–2013	0.97 ± 0.02	60.69	< 0.001
	C. Crozier	2014–2017	1.08 ± 0.02	5.35	< 0.001
4	C. Royds	2002–2013	0.87 ± 0.04	23.69	< 0.001
	C. Royds	2014–2017	0.75 ± 0.06	− 2.21	0.03

Models 3 and 4 are comparing differences between the study period (2014–2017) and the longer-term mean at the same colony.

### Subcolony characteristics

**Figure 3:**
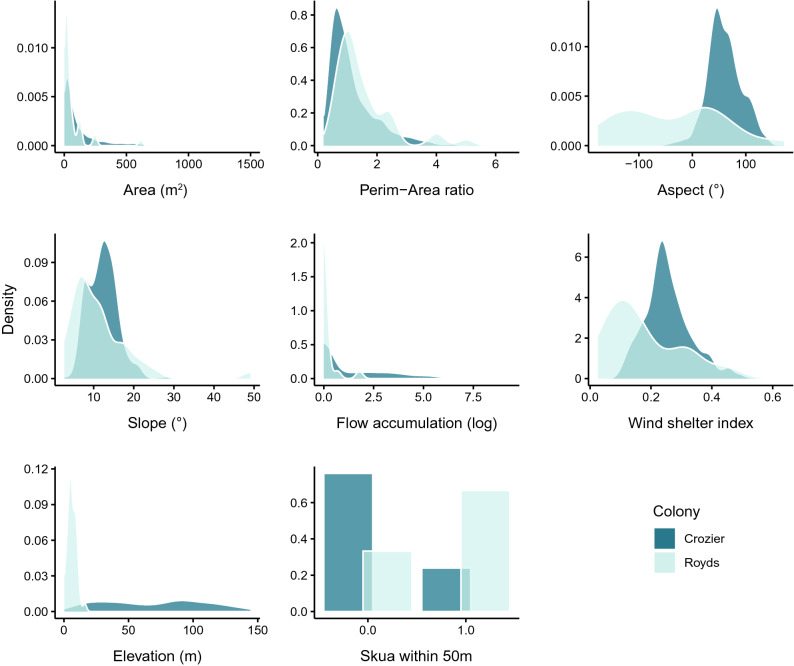
Distributions of spatial habitat covariates for all subcolonies at Cape Crozier and Cape Royds, Ross Island, Antarctica. Means of all covariates were statistically different between the two colonies. Last panel indicates the number of subcolonies that have at least one skua nest within 50 m (0 = no skua nest, 1 = skua nest within 50 m). The distribution of subcolony areas at Cape Crozier had an extremely long tail so 21 (out of 723) subcolonies with areas greater than 1500 m^2^ were excluded from this figure to aid visualization. The max subcolony area at Cape Crozier was 9568 m^2^.

The range and distribution of the spatial habitat covariates that we assessed differed between the two colonies (Fig. 3). Cape Crozier subcolonies were significantly higher in elevation, larger, had higher flow accumulation, and were more sheltered from the wind compared to Cape Royds (Mann–Whitney U tests for non-parametric data, p < 0.05; Fig. 3). The average perimeter-to-area ratio was significantly higher at Cape Royds, indicating a higher proportion of edge nests compared to interior nests (Fig. 3). Slope aspect also differed significantly (Watson’s two sample test statistic 2.0353, p < 0.001), with the average subcolony at Cape Crozier on slopes facing east-northeast (circular mean = 60.1°) while the slope aspect of Cape Royds subcolonies was more variable with peaks at ~ 20° and − 110° with the average slope facing northwest (circular mean = − 54.9°; Fig. 3). At Cape Crozier only ~ 1 in 4 subcolonies had a skua nest within 50 m (24.6%), whereas more than half of the subcolonies at Cape Royds had a skua nest nearby (68.8%, Chi-squared = 41.91, p <  < 0.001). Finally, flow accumulation at Cape Royds was very minimal, only 3 study subcolonies had non-zero estimated surface flow and surface flow at all 3 was very low. For this reason, we did not include flow accumulation as a covariate in the models for Cape Royds. We also note that the largest subcolonies present at either colony were too large to count from the ground, so our sample does not include the full range of subcolony sizes present (supplemental Figs. 1 and 2).

### Modeling subcolony quality

**Figure 4:**
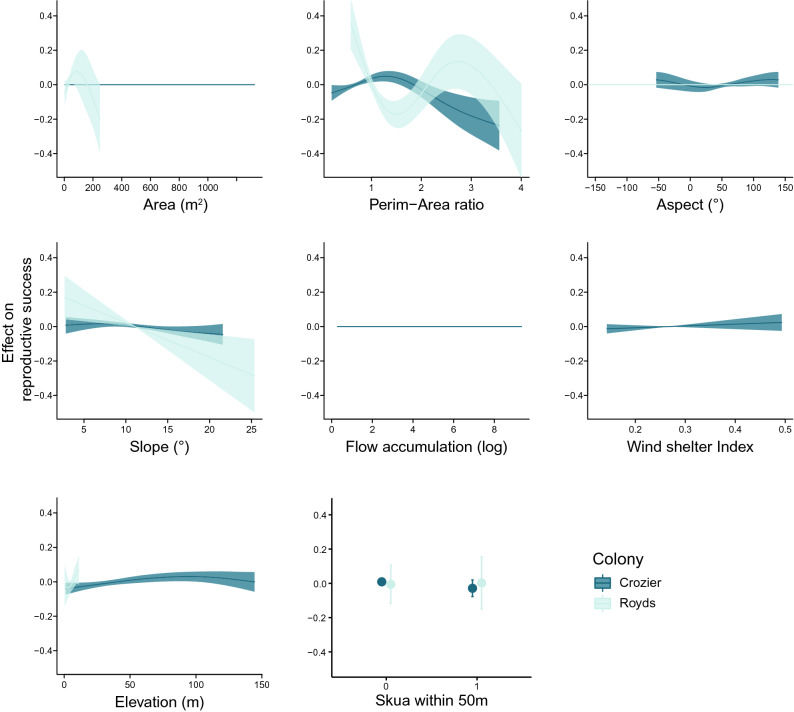
Modeled relationships (using generalized additive models) between nesting habitat characteristics and reproductive success (chicks per pair) of Adélie penguins at Cape Crozier and Cape Royds, Ross Island, Antarctica, 2014–2017. Effects are relative to mean reproductive success.

The top-ranked model explaining spatial variability in subcolony reproductive success at both colonies was the model that did not include the interaction with year. The model with year interactions was competitive at Cape Crozier (ΔAICc = 2, Table 3). The top model for Cape Royds was the model that included aspect instead of the wind shelter index. Overall, habitat covariates explained much less of the spatial variability in subcolony reproductive success at Cape Crozier compared to Cape Royds (adjusted R^2^ = 0.12 at Cape Crozier vs. adjusted R^2^ = 0.50 at Cape Royds). The correlation between observed and predicted reproductive success was r = 0.38 for Cape Crozier (p < 0.001) and r = 0.74 for Cape Royds (p < 0.001). The most important variable at both colonies was perimeter-to-area ratio and the estimated percent decrease in R^2^ when perimeter-area ratio was removed was similar, 46.0% at Cape Crozier compared to 41.2% at Cape Royds (Table 4). Elevation was the second most important variable at Cape Crozier but was much less important at Cape Royds (ΔR^2^ = 13.6% at Crozier and 2.4% at Cape Royds). At both colonies, reproductive success tended to increase with elevation, although it started to decline again at the highest elevations at Cape Crozier (Fig. 4). At Cape Royds, slope and area were about equally important (ΔR^2^ = 7.8% and 7.2% respectively; Table 4) with both showing a negative relationship to reproductive success. Slope was the 3rd ranked variable at Cape Crozier (ΔR^2^ = 11.1%, also a negative trend) and aspect was the 4th (ΔR^2^ = 9.4%). But both effects were very small with 95% confidence intervals completely encompassing a line with a slope equal to zero. Aspect was dropped from the Cape Royds model (Table 4). Finally, wind shelter was the lowest ranked smooth retained in the Cape Crozier model (ΔR^2^ = 2.6%). Wind shelter showed a weak positive relationship with reproductive success at Cape Crozier but again the confidence intervals encompassed a line with zero slope. Wind shelter was not in the top Royds model. Being near a skua nest made no difference at either colony (Fig. 4).

**Table 3:** Generalized additive models of spatial variability in reproductive success for Cape Crozier and Cape Royds, Ross Island, Antarctica, 2014–2017.

Model	Description	LogLik	AICc	ΔAICc	df	Weight	n	R^2^ (adj)
**Cape Royds**								
m2	Base (-wind shelter)	15.8	− 8.3	0.00	10.1	1	86	0.50
m3	m2 × year	16.0	10.3	18.6	16.8	< 0.001	86	0.46
m1	Base (-aspect)	6.2	11.0	19.3	10.2	< 0.001	86	0.38
**Cape Crozier**								
m1	Base	167.0	− 307.7	0.0	12.5	0.73	292	0.12
m2	m1 × year	185.5	− 305.7	2.0	29.2	0.27	292	0.18

The base model was a model that initially (prior to automatic variable selection) included all covariates but no year interactions. Covariates are listed below the table. Models were ranked and weighted by Akaike’s information criterion corrected for small sample sizes (AICc; n/df < 40).

Base model: area + perimeter-area ratio + aspect + slope + flow accumulation + wind shelter index + elevation + skua within 50 m.

Year interaction model : area + perimeter-area ratio + (aspect × year) + (slope × year) + (flow accumulation × year) + (wind shelter index × year) + (skua × year).

**Table 4:** Variable importance for each covariate in the top model at Cape Crozier and Cape Royds, Ross Island, Antarctica, 2014–2017.

Term removed	*Cape Crozier*			*Cape Royds*		
	R^2^ (adjusted)	ΔR^2^	%ΔR^2^	R^2^ (adjusted)	ΔR^2^	%ΔR^2^
None (top model)	0.12	0	0	0.50	0	0
Area	NA	NA	NA	0.46	0.036	7.2
Perim-area ratio	0.06	0.054	46.0	0.29	0.205	41.2
Aspect	0.11	0.011	9.4	NA	NA	NA
Slope	0.10	0.013	11.1	0.46	0.039	7.8
Wind shelter	0.11	0.003	2.6	NA	NA	NA
Elevation	0.10	0.016	13.6	0.49	0.012	2.4
Skua within 50 m	0.11	0.003	2.6	0.50	− 0.006	+ 1.2

The ΔR^2^ is the difference between the adjusted R^2^ of the top model and the adjusted R^2^ of the top model with the variable of interest removed. %ΔR^2^ is the percent decrease in the original R^2^ when that variable was removed. The plus sign for the skua proximity variable at Cape Royds indicates a slight increase in the adjusted R^2^ when that variable was removed. Flow accumulation does not appear in the table because it was not included in either top model.

The shape of the smoothed curves differed between colonies, sometimes dramatically. For perimeter-to-area ratio in particular, the relationship at Cape Crozier was peaked, with maximum reproductive success between ratios of 1 and 2 (Fig. 4). At Cape Royds, the smoothed curve resembled a cubic relationship with a maximum at the lowest values, and a minimum between ratios of 1 and 2 (similar values as the maximum at Cape Crozier) and at maximum values (ratios near 4; Fig. 4). The relationship with subcolony area was also very different, it was dropped from the Cape Crozier model while the relationship at Cape Royds indicated a steep decline in success for the larger subcolonies, although the confidence interval indicated high uncertainty around the shape of this smooth.

**Figure 5:**
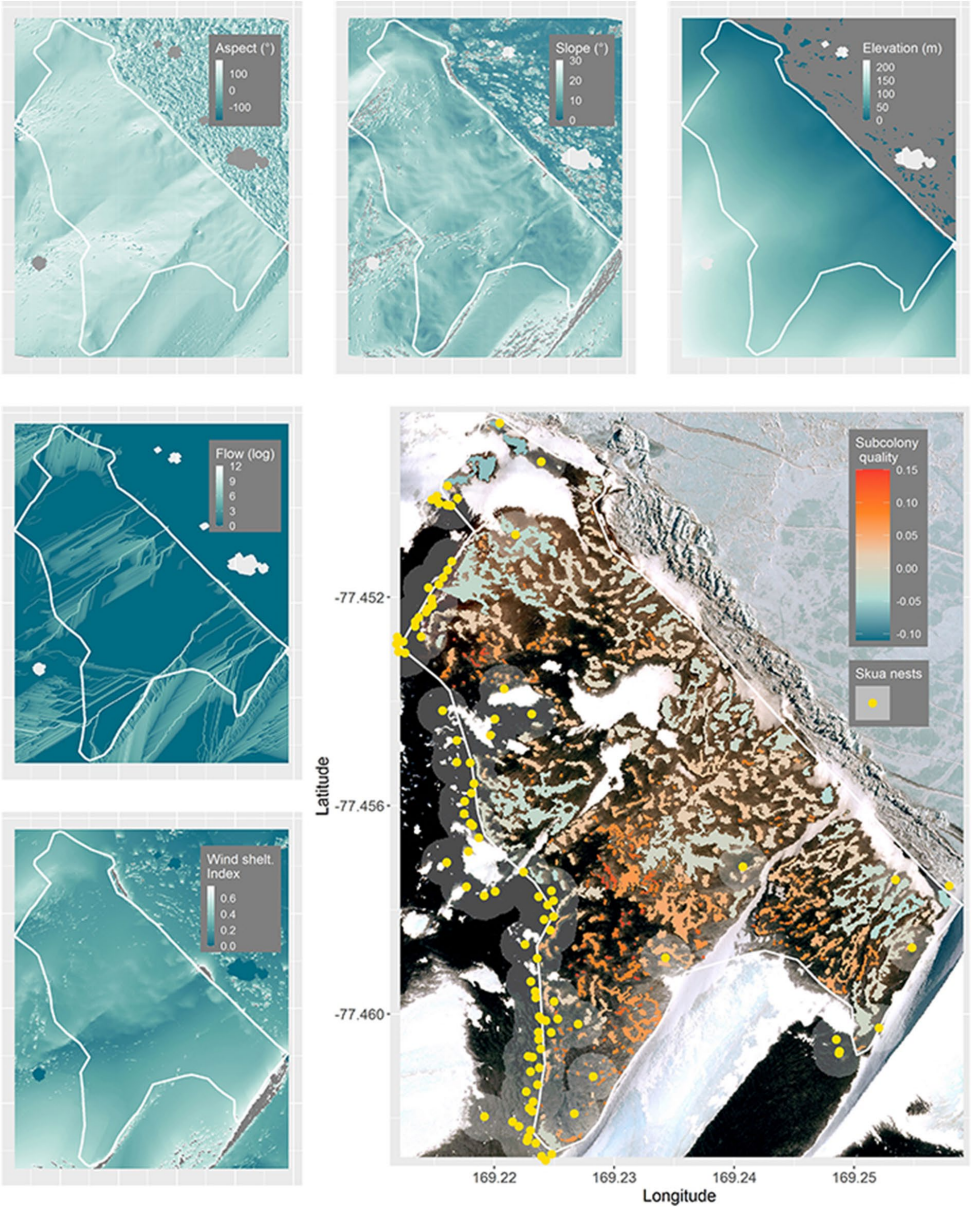
Spatial habitat covariate layers and predicted subcolony quality at Cape Crozier, Ross Island, Antarctica (background WorldView-3 satellite image, November 20, 2014, copyright 2014 DigitalGlobe, NextView License). Positive values (orange) indicate above average subcolony quality and negative values (teal) indicate below average subcolony quality relative to the mean at Cape Crozier. Yellow points indicate locations of skua nests included in the model and grey circles show the 50 m radius used to indicate skua nest proximity.

**Figure 6:**
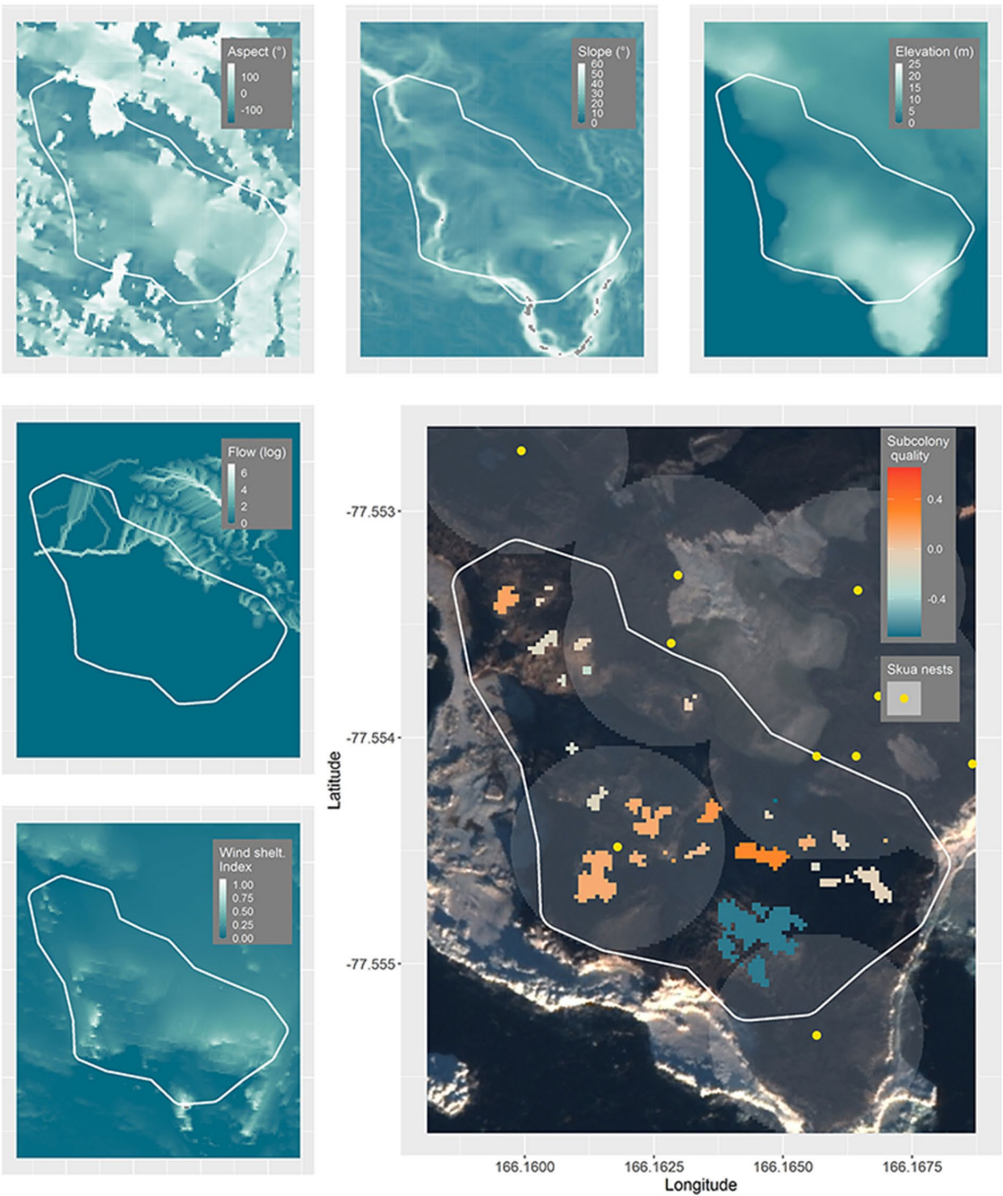
Spatial habitat covariate layers and predicted subcolony quality at Cape Royds, Ross Island, Antarctica (background WorldView-2 satellite image, November 27, 2015, copyright 2015 DigitalGlobe, NextView License). Positive values (orange) indicate above average subcolony quality and negative values (teal) indicate below average subcolony quality relative to the mean at Cape Royds. Yellow points indicate locations of skua nests included in the model and grey circles show the 50 m radius used to indicate skua nest proximity. To aid visualization, one subcolony with very low predicted quality was not included on the color scale and appears as a small grey pixel in the Northwest section of the colony. This subcolony had the lowest predicted quality of − 1.13.

When subcolony quality was predicted spatially at each colony based on the top model, at Cape Crozier, low quality subcolonies occurred on the edges of the colony, including both low elevation subcolonies near the shore (ice or open water), and high elevation subcolonies near skua nests and snowbanks (Fig. 5). At Cape Royds, one of the lowest predicted quality subcolonies was also the largest (Fig. 6). The range of predicted subcolony quality was much narrower at Cape Crozier, − 0.43 to 0.15 but with the majority between − 0.10 and 0.15, with zero representing the average subcolony quality (i.e., reproductive success) for the colony. Predicted subcolony quality at Cape Royds was much more variable (range − 1.13 to 0.30) but with the majority > − 0.4.

## Discussion

The vast majority of Adélie penguin colonies in Antarctica are relatively small, with nearly 60% similar in size to Cape Royds^4,31,32^. Only seven colonies are > 100,000 pairs and only 3 other colonies are of similar size to Cape Crozier^31,33^. For the Cape Crozier colony to have achieved this unusual size, it is reasonable to hypothesize that it contains higher than average quality terrestrial nesting habitat in addition to being located near high quality foraging habitat. Reproductive success varied spatially with subcolony characteristics at both colonies, but the total spatial variance was much less at Cape Crozier and subcolony quality appeared to be more influential at Cape Royds. Higher average reproductive success along with lower spatial and interannual variability at Cape Crozier supports the hypothesis that the nesting habitat at Cape Crozier is more homogenous and of higher overall quality. Models for the two colonies had some variables in common, e.g., at both colonies, penguins nesting in subcolonies that had lower perimeter-to-area ratios, shallower slopes, and were higher in elevation, tended to be more successful at raising chicks. However, relationships between reproductive success and habitat covariates were not identical at the two colonies, and some variables appeared to influence subcolony quality at one colony but not the other (e.g., aspect at Cape Crozier and subcolony area at Cape Royds). Hence, there does not appear to be a universal definition of high-quality habitat that fits both colonies.

The most influential variable at both colonies was perimeter-to-area ratio: the subcolonies with the highest perimeter-to-area ratios (higher proportion of edge nests), had lower reproductive success than those with a low perimeter-to-area ratio (low proportion of edge nests). On average, the perimeter-to-area ratio at Cape Royds was higher than at Cape Crozier (Fig. 3), indicating the overall proportion of edge nests at Cape Royds was higher. Although the shape of the estimated smooths differed, at both colonies the highest perimeter-to-area ratios were associated with the lowest reproductive success. Although proximity to a skua nest was not an important factor, the negative effect of a high perimeter-to-area ratio implicates nest predation as a dominant force driving spatial variation in reproductive success^27,32,58,67,68^. Skuas primarily attack from the ground and target edge nests more frequently^27^. While skuas may defend foraging areas around their nest sites, they are not restricted to foraging only near their nests and not all skuas foraging in the colony have nesting territories, so although we hypothesized there might be a difference for subcolonies that were close to nests, it does not necessarily have a direct correlation to predation pressure for an area. Some areas that are outside of a skua nesting territory may in fact have higher predation pressure because there is no territorial skua to chase others away^59^.

Removing perimeter-to-area ratio from the models resulted in a larger absolute difference in the variance explained at Cape Royds (ΔR^2^ = 0.21 at Cape Royds vs. 0.05 at Cape Crozier, Table 4), suggesting that the influence of skuas on penguin reproductive success is higher at Cape Royds. Indeed, the number of skua nests has been shown to have a log-linear relationship with Adélie penguin numbers^69^ with higher relative densities of skuas at small colonies. At Cape Crozier there are an estimated 1099–1347 nesting skua pairs (with ~ 300,000 penguin pairs or approximately 0.004 skua pairs per penguin pair) compared to 45 pairs of skuas per 3000 penguin nests at Cape Royds (0.015 skua pairs per penguin pair, nearly 4 times the density^69^. It is also possible that there are other variables that we did not measure that correlate with perimeter-area-ratio and nest success, such as breeding experience.

In recent decades, prior to 2001, Cape Royds had been relatively stable with the number of breeding pairs fluctuating around 3500, possibly indicating a population near carrying capacity or limited by other external factors (e.g. lower overwinter survival^8,27,43,70^). In 2001, the mega iceberg B-15 broke off from the Ross Ice Shelf and lodged along the north shore of Ross Island, causing extensive sea ice to remain in place and discouraging travel between open water and the colony^71,72^. This event led to a sharp decrease in colony size: the number of breeding pairs was abruptly reduced to less than half of immediately preceding years^8^. Access to the colony was restored in 2005^72^ but the population has been slow to recover from this setback and has yet to reach the former high count (the most recent estimate was 2330 pairs in 2019; Ballard and Schmidt unpublished data). In contrast, Cape Crozier has grown rapidly since the iceberg and is now well beyond the hypothesized size limit imposed by energetic constraints^8,73^. The sudden decrease in colony size at Cape Royds was likely accompanied by increased fragmentation of subcolonies^74–76^ and an associated increase in the relative proportion of edge nests, driving a decline in subcolony quality and perhaps allowing increased skua predation to hinder recovery. High perimeter-area-ratios and slow growth at Cape Royds are consistent with a population “frozen” in a suboptimal arrangement of nests^74,75^. Using an individual-based model, McDowall and Lynch^75^ demonstrated that even when penguins are “allowed” to occasionally change nest sites, the slow rate of movement resulting from high nest site fidelity and incomplete information leads to suboptimal nest arrangements: decisions about when and where to move are made by individuals with incomplete information as the quality of their final nest site depends on unknown movements of other penguins. Thus, nest site fidelity, even if incomplete, can result in a persistently fragmented configuration of nests that inhibits growth.

Aspect, and the related snow drift risk, has been shown to be an important variable influencing reproductive success of Adélies at several colonies on the Antarctic Peninsula^38,40^. In contrast, aspect was not a strong predictor of reproductive success in our study: it was the 4th most important variable at Cape Crozier but the 95% confidence interval encompassed a line with zero slope so the effect is likely not significant and was not retained in the top Cape Royds model. We used a wind shelter index to assess snow drift risk directly because it includes the influence of the surrounding terrain in addition to the aspect of the subcolony itself. Wind shelter was higher on average at Cape Crozier and showed a weak positive relationship with subcolony reproductive success, possibly indicating higher shelter/snow drift risk was associated with higher success, but again the effect was weak with confidence intervals encompassing a line with slope of zero so the significance is uncertain. Wind shelter was not included in the top Cape Royds model. The dominant aspect at the two colonies differs substantially, with the slope at Cape Crozier oriented primarily facing northeast away from the dominant wind direction, while Cape Royds is mostly on a plateau, with proportionally more subcolonies oriented south but with more variability within the colony (Fig. 3). Cape Royds also has much less elevational change (0 to ~ 20 m compared to 0 to ~ 150 m at Cape Crozier; Figs. 3, 5, and 6), so snow drift risk is relatively low for all subcolonies at Cape Royds, no matter their aspect because there are fewer terrain features to create snow drifts. In comparison, the terrain at Cape Crozier is much more variable and extreme, with several large ridges and hills that generate large semi-permanent snow drifts within the colony (Fig. 5). Nonetheless, aspect and wind shelter each explained very little of the total variation and did not appear to be important drivers of spatial variation in habitat quality at Cape Crozier.

The effect of wind, snow, and flooding are likely to vary, depending on the storms and weather experienced at the colony in any given year. The fact that the model with year interactions was competitive for Cape Crozier supports this idea. The wind shelter variable is particularly sensitive to the selection of the dominant wind direction. Although we tried to account for this uncertainty by allowing some tolerance around the dominant wind direction, that might not accurately capture the dynamic nature of storms which can vary in wind direction, wind speed, and amount of snow deposited^61^. The relatively short duration of the study, as well as the lack of direct quantitative information on snowfall in our study area, limited our ability to examine the true interaction between terrain attributes, wind, and air temperature. If examined over a longer time frame, the relationship between wind, snow-drift, and flood risk, may turn out to be more important than it appeared during the 4 years of this study^see e.g.^^38,41^.

Interestingly, subcolony size was not a predictor of reproductive success at Cape Crozier, but was the 3rd ranked variable at Cape Royds. Higher quality subcolonies might be expected to be larger because successful breeders may attract recruits to settle nearby (e.g., the public information hypothesis)^77,78^. This did not appear to be the case at either colony, with some of the largest subcolonies having the lowest predicted habitat quality (e.g., the largest subcolony at Cape Royds in Fig. 6). Notably, the largest subcolonies could not be counted at either colony so were not included when fitting the model. Thus, many of the large subcolonies fall outside the range of the measured response and predictions for those subcolonies should be viewed with appropriate caution.

## Conclusion

The quality of nesting habitat appears to play a role in determining subcolony reproductive success on Ross Island, but the importance can vary between colonies. The most important factor at both colonies studied here, perimeter-to-area ratio, was not a terrain attribute, but an emergent characteristic of subcolony nesting aggregations. Because the majority of Adélie penguin colonies are small, a substantial fraction of known breeding locations are likely to experience disproportionate impacts from nest predators and stochastic events that drive fragmentation. As colonies grow, the average perimeter-to area ratio should decline, increasing subcolony quality and providing a positive feedback mechanism (or Allee effect) to reinforce growth. But if the frequency of fragmenting events increases under climate change, it could result in fewer colonies reaching the threshold where they can escape the predator trap. Although small colonies dominate the global distribution of colony sizes, the sum of all the colonies with less than 3000 pairs together contains fewer breeding pairs (~ 100,000 pairs) than Cape Crozier on its own and < 5% of the global breeding population in total^31^. Thus, the vast majority of Adélie penguins nest in larger aggregations and may be less susceptible to the effects of fragmentation and predation. Our results emphasize the challenges faced by small colonies and highlight the importance of protecting larger colonies from human impacts (see also^33^), as well as gaining a better understanding of the specific factors driving nest predation at a broader range of colony sizes. The effects of most terrain covariates appeared to be less important than the edge effects of perimeter-to-area ratio, but there are several caveats to these results. Additional effort should be directed at better quantification of how episodic weather interacts with fine-scale terrain as well as assessing the reproductive success of the largest subcolonies.

## Supplementary Information

Supplementary Information.

## Data Availability

Data collected are available at California Avian Data Center (CADC) hosted by Point Blue Conservation Science (http://data.prbo.org/apps/penguinscience/) and the US Antarctic Program Data Center (https://www.usap-dc.org/).
