# Supplementary material for: The influence of subcolony-scale nesting habitat on the reproductive success of Adélie penguins
Source: Sci Rep. 2021 Jul 28;11:15380. doi: 10.1038/s41598-021-94861-7 (PMC8319293; doi:10.1038/s41598-021-94861-7)
Supplement: Supplementary file 1 — Supplementary Information. [file 41598_2021_94861_MOESM1_ESM.pdf]

The influence of subcolony-scale nesting habitat on the reproductive success of Adélie penguins

Annie E. Schmidt, Grant Ballard, Amélie Lescroël, Katie M. Dugger, Dennis Jongsomjit, Megan L. Elrod, David G. Ainley

Supplemental Figures

Figure S1. Distributions of spatial habitat covariates for all (dark) and sample (light) subcolonies at Cape Crozier, Ross Island, Antarctica. Last panel indicates the number of subcolonies that have at least one skua nest within 50m (0= no skua nest, 1=skua nest within 50m). The distribution of subcolony areas at Cape Crozier had an extremely long tail so his hard to visualize the differences. The max subcolony area at Cape Crozier is 9568 m^2 whereas the max subcolony area in the sample was 1325 m^2.

Figure S2. Distributions of spatial habitat covariates for all (dark) and sample (light) subcolonies at Cape Royds, Ross Island, Antarctica. Last panel indicates the number of subcolonies that have at least one skua nest within 50m (0= no skua nest, 1=skua nest within 50m).

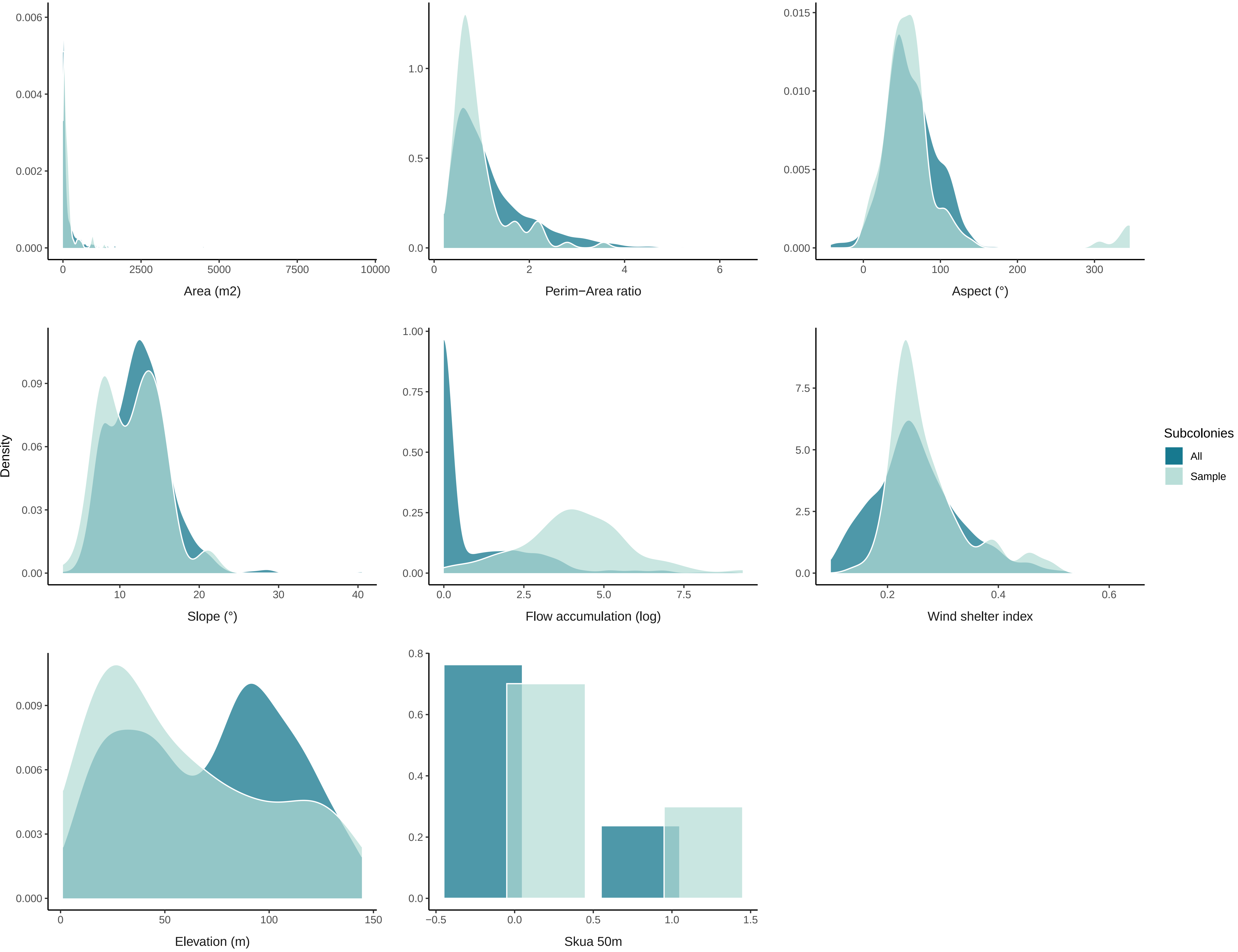

Area (m2)
Perim−Area ratio
Aspect (°)
Slope (°)
Flow accumulation (log)
Wind shelter index
Elevation (m)
Skua 50m
Density
Subcolonies
All
Sample

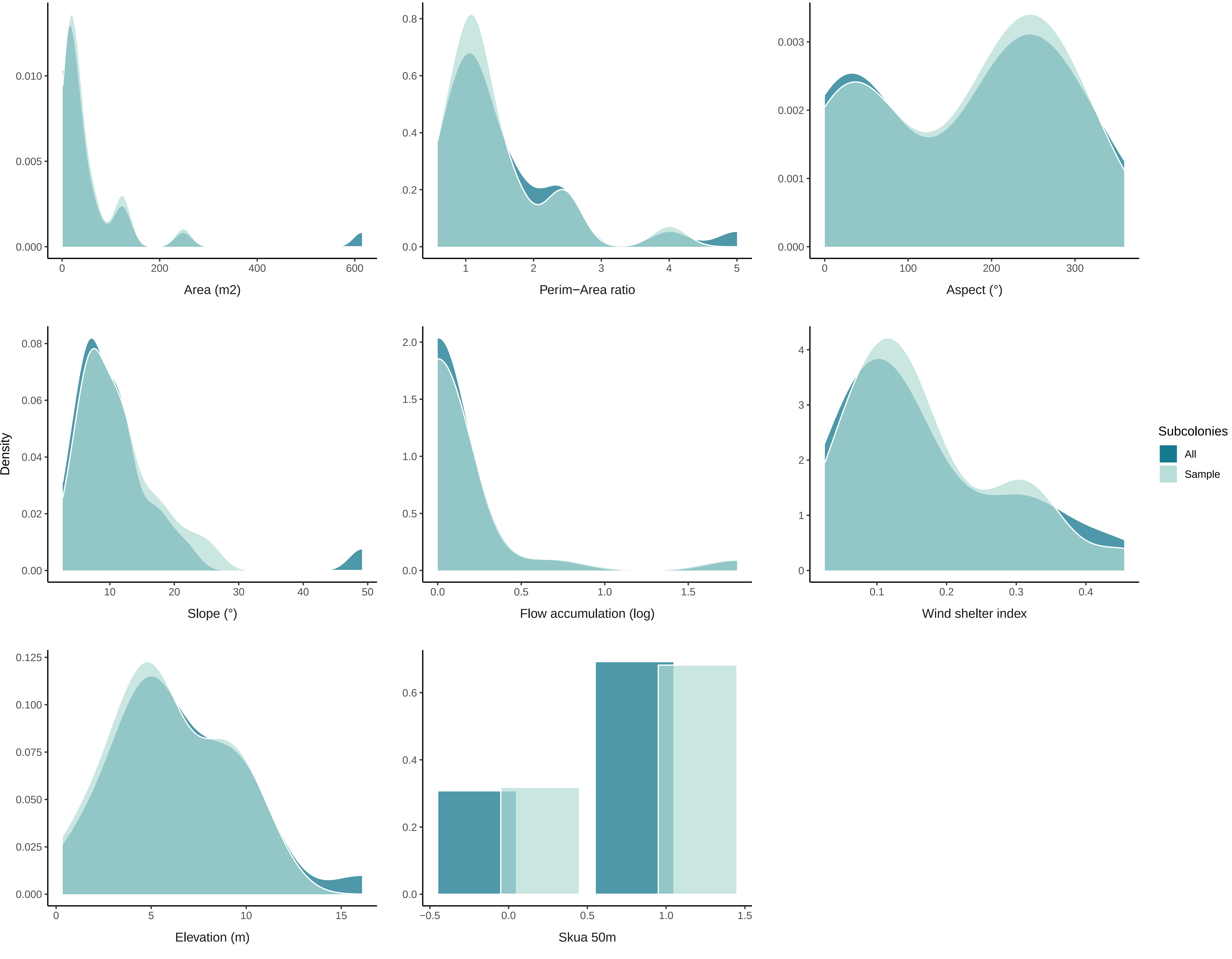
Density
Area (m2)
Perim−Area ratio
Aspect (°)
Slope (°)
Flow accumulation (log)
Wind shelter index
Elevation (m)
Skua 50m
Subcolonies
All
Sample
